# Use of trained African giant pouched rats as a predictor of clinical diagnosis of pulmonary TB

**DOI:** 10.5588/pha.25.0010

**Published:** 2025-09-03

**Authors:** J. Soka, S. Mwimanzi, C.D. Fast, G. Mwesiga, N. Edward, M. Stephen, R. Kondo, C. Cox, N. Beyene, T.B. Agizew

**Affiliations:** ^1^ Anti-Persoonsmijnen Ontmijnende Product Ontwikkeling (APOPO), Tuberculosis Department, Sokoine University of Agriculture, Morogoro, Tanzania;; ^2^ Department of Biology, University of Antwerp, Belgium;; ^3^ APOPO TB Research Project, Armauer Hansen Research Institute (AHRI), Addis Ababa, Ethiopia.

**Keywords:** tuberculosis, Tanzania, Ethiopia, smear negative, *Cricetomys ansorgei*

## Abstract

For over a decade, trained African giant pouched rats have been employed in detecting missed pulmonary TB (PTB). However, the relationship between rat-positive results and subsequent clinical PTB or extrapulmonary TB (EPTB) has not been previously reported. This report highlights the use of rat-positivity as a predictor for PTB clinical diagnosis and treatment among presumptive TB. Treating physicians were 1.39 times more likely to clinically diagnose and treat rat-positives than rat-negatives: 12% versus 9%, respectively, odds ratio=1.39, 95% confidence interval: 1.05–1.84*.* No difference was observed among EPTB.

In 2023, more than 10.8 million people contracted TB with 1.25 million deaths,^1^ and TB remains a significant public health problem in sub-Saharan Africa. Although TB is declining by 5% on average annually in Tanzania and Ethiopia,^2^ considerable gaps remain in finding and treating cases, and a decline of just 5% per year presents a challenge to achieving the End TB targets. One approach to improving TB diagnosis is the use of African giant pouched rats (*Cricetomys ansorgei*), trained by Anti-Persoonsmijnen Ontmijnende Product Ontwikkeling (APOPO). The use of rats to detect TB by smell has been well documented, including a meta-analysis.^3–5^ Previous studies that used APOPO-trained rats reported an average annual increase in TB detection of 40% over sputum-smear microscopy in Tanzania.^3,4^ Following sniffing by trained rats, rat-positive samples were verified by concentrated sputum Ziehl-Neelsen smear microscopy (CS-ZN). However, if culture (gold standard), or Xpert MTB/RIF Ultra, or TrueNat, all with better sensitivity than smear or Xpert MTB/RIF, were used as confirmatory tests, the added value of rats might have been higher. For example, despite a rat-positive result indicating TB, if the confirmatory tests were bacteriologically negative, the treatment decision would be left to the discretion of the treating physicians. Patients identified as having TB by rats but bacteriologically negative have not been consistently studied and reported before. Assessing rat-positivity as a predictor of a clinical diagnosis of TB may add value to the treating physician’s decision making. The aim of this study was therefore to evaluate whether rat-positive results are good predictors of a PTB diagnosis. We did this by comparing clinically diagnosed and treated TB patients with those who were rat-positive and those who were not.

## METHODS

APOPO’s model was second-line TB screening by rats among presumptive TB patients deemed negative by routine Directly Observed Therapy Short course (DOTS) facilities screening. In the past decade, molecular WHO-recommended rapid diagnostic (mWRD) tests have been used to replace traditional methods such as smear microscopy. People diagnosed using mWRD, smear, or culture are defined as being ‘medically diagnosed’ TB, and those without confirmation are classified as ‘clinically diagnosed’ TB.^7^ In 2023, globally 38% of reported TB cases were clinically diagnosed.^7^ Since 2017, APOPO has been re-evaluating samples from smear or Xpert-negative presumptive TB patients using rats; and rat-positive samples are confirmed by CS-ZN microscopy, yielding an annual average of 40% in case detection.^3^

In this study, between 1^st^ January to 31^st^ December 2023, second sputum samples from Mbagala Rangi Tatu hospital’s presumptive TB patients, who tested negative by smear or Xpert, were re-evaluated by rats. The APOPO TB detection model, sample collection, and re-evaluation procedures are described elsewhere.^6^ An average of five rats were used to re-evaluate the samples. Rat-positive (indicated positive by ≥1 rat) were verified using CS-ZN microscopy, and CS-ZN-confirmed results were reported back to the DOTS facilities for treatment. The clinicians involved in clinically diagnosing TB were blinded to the rats’ results. By comparing clinically diagnosed and treated TB patients with those who were rat-positive and those who were not, we use a retrospective design to assess whether rat-positivity is a good predictor for PTB diagnosis.

## RESULTS

A total of 2,401 patients were identified as smear- or Xpert-negative presumptive TB using ZN or Xpert MTB/RIF: and 49% (1,172/2401) were male. The median age was 38 years (interquartile range, 25–51) years). Children and adults represented 9% (217/2,401) and 87% (2,091/2,401), respectively. Among the 2,401 smear or Xpert-negative patients, 1,031 and 1,370 were rat-positive and rat-negative, respectively. Of those that were rat-positive, 14% (144/1031) were confirmed positive and 86% (887/1031) were negative by CS-ZN. Of the 887, 102 and 41 were clinically diagnosed as PTB and extrapulmonary TB (EPTB), respectively, whereas among the rat-negatives, 117 and 66 were PTB and EPTB, respectively (see Figure). Clinical PTB among rat-positives and rat-negatives was 12% (102/846) and 9% (117/1304), odds ratio=1.39, 95% confidence interval: 1.05–1.84. For rat-positives and rat-negatives, the likelihood for EPTB clinical diagnosis was similar: 5% (41/785) and 5% (66/1253), *p*=0.965 (Table).

**FIGURE.**
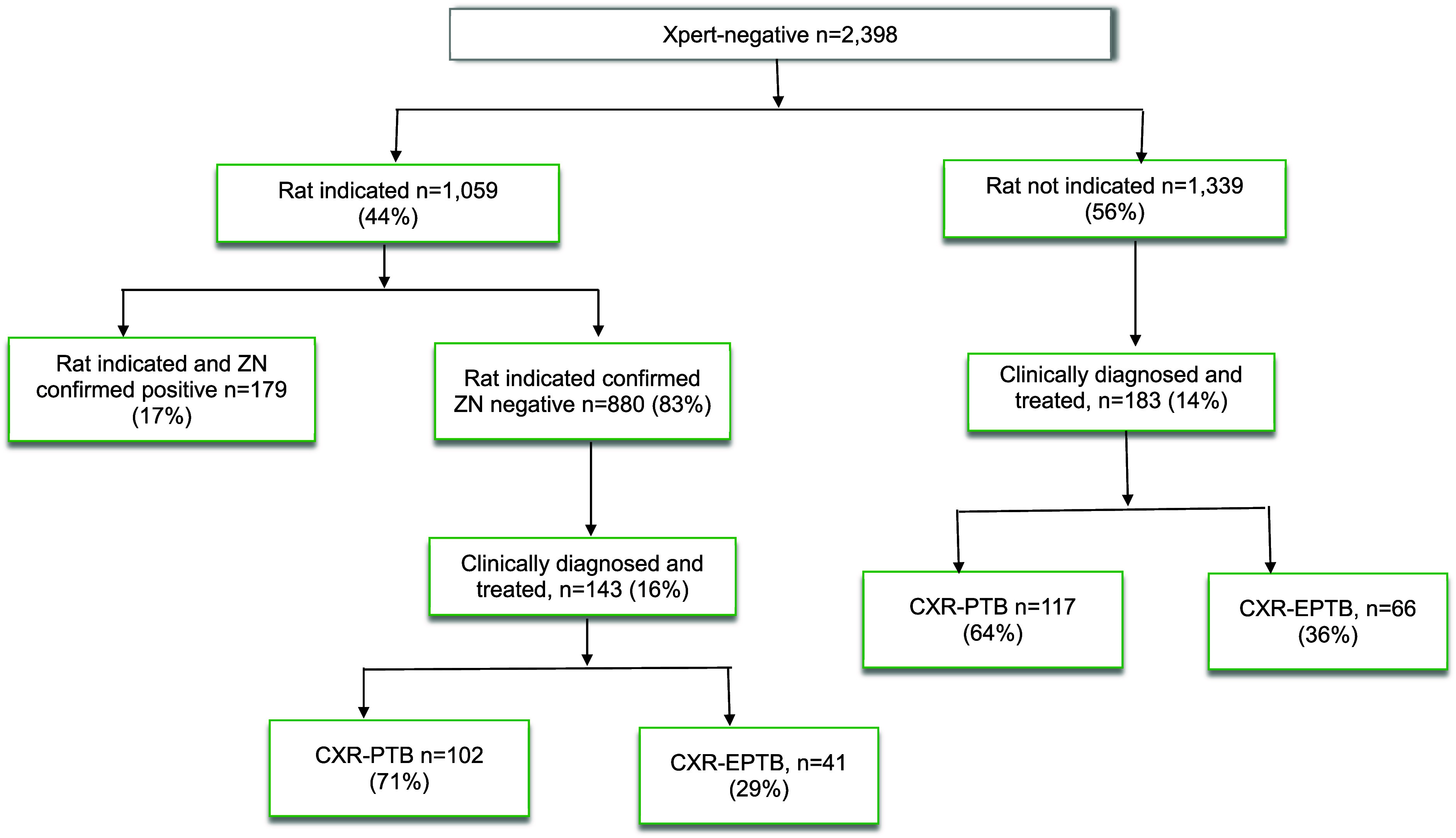
Rat indication and clinical diagnosis of PTB and EPTB. TB identified by detection rats at APOPO and confirmed by concentrated Ziehl-Neelsen (ZN) sputum smear microscopy. CXR = chest x-ray; PTB = pulmonary TB; EPTB = extrapulmonary TB.

**TABLE.** Clinical diagnosis of pulmonary TB among rat indicated and not indicated patients.

	Rat-indicated/AFB negative	Rat not indicated	OR	95% CI	P value
Clinical TB	143 (16%)	183 (13%)	1.25	0.98–1.56	0.068
Not TB	744 (84%)	1187 (87%)			
Total	887	1370			
Clinical PTB	102 (12%)	117 (9%)	1.39	1.05–1.84	0.021
Not TB	744 (88%)	1187 (91%)			
Total	846	1304			
Clinical EPTB	41 (5%)	66 (5%)	0.99	0.67–1.48	0.965
Not TB	744 (95%)	1187 (95%)			
Total	785	1253			

AFB = acid fast bacilli; OR = odds ratio; CI = confidence interval; PTB = pulmonary TB; EPTB = extrapulmonary TB.

Clinical PTB among rat-positives and rat-negatives among children (<15 years old) was 9% (9/101) and 16% (18/112), P=0.112; Male versus female was 52% (53/102) and 65% (76/117), p=0.051; and HIV-infected and non-infected was 24% (24/102) and 27% (31/117), p=0.614, respectively.

## DISCUSSION

To our knowledge, this is the first report on the predictive value of use of rats for the clinical diagnosis of PTB. The odds of being clinically diagnosed with PTB among rat-positive individuals were 39% higher compared to rat-negative, underscoring the value of rat-based detection in prioritizing individuals for further clinical evaluation.

Within the context of APOPO, using a more sensitive diagnostic test as a confirmatory test is worth considering. Thus far, APOPO is not widely using molecular tests for verification for a logistical reason. From literature, even Xpert does not seem to have that high sensitivity among smear-negative samples. A systematic Cochrane review showed that among smear-negative samples, the Xpert has a pooled sensitivity of 61-67%.^7,8^ Therefore, Xpert Ultra or TrueNat might be better options.

Globally, the high rate of clinical diagnosis of TB remains a concern and in 2023, it was reported to be 38%.^4^ Literature suggests that TB treatment outcome, especially mortality, is higher among clinically diagnosed TB versus bacteriologically confirmed TB.^9^ There is therefor a need to increase the percentage of people diagnosed with PTB on bacteriological confirmation.^9^ Therefore, if resources allow, we recommend that APOPO is combined with more sensitive diagnostic tests such as mWRD. Understandably, cost remains a concern, and the recently used pooled (combining samples) testing approach might help to reduce cost.^10^

## CONCLUSION

Demonstrating rat-positivity as a predictor for clinical diagnosis of PTB is an exciting new prospect. In settings with limited laboratory capacity, despite a negative CS-ZN confirmatory test, informing clinicians of rat-positives has a potential for enhancing the diagnosis of bacteriologically negative PTB. The rats’ value for clinical diagnosis warrants further investigation.
